# The Role of Trace Amine-Associated Receptor 1 (TAAR1) in the Pathophysiology and Treatment of Depression

**DOI:** 10.2174/011570159X370669250526115723

**Published:** 2025-06-23

**Authors:** Wei Guan

**Affiliations:** 1 Department of Pharmacology, Pharmacy College, Nantong University, Nantong, 226001, Jiangsu, China

**Keywords:** Depression, TAAR1, antidepressant, ulotaront, neuropsychiatric disorders, signalling pathway

## Abstract

Depression is a chronic and recurrent psychiatric condition believed to result from an interaction between genetic susceptibility and environmental stimuli. Although current therapies prescribed for depression can be effective, it will take several weeks to demonstrate their full effectiveness and is often accompanied by side effects and withdrawal symptoms. In this regard, the discovery of new antidepressant drugs with unique, higher curative effects and fewer adverse reactions is the pursuit of pharmaceuticals. Trace amine-associated receptor 1 (TAAR1), a G-protein coupled receptor (GPCR) that is broadly expressed in the mammalian brain, especially within cortical, limbic, and midbrain monoaminergic regions and activated by “trace amines” (TAs). It is allegedly involved in modulating dopaminergic, serotonergic, and glutamatergic transmission, which makes TAAR1 a new drug target for the treatment of dysfunction of monoamine-related disorders. Moreover, TAAR1 agonists have attracted interest as potential treatments for depression due to their role in regulating monoamine neurotransmission. In fact, Ulotaront (a TAAR1 agonist) is reported to be currently undergoing phase 2/3 clinical trials in order to test its safety and efficacy in the treatment of major depressive disorder (MDD). However, the final results of this Phase 2/3 clinical study have not been announced yet, and the efficacy and safety of Ulotaront in the treatment of depression still need further observation and research. Thus, this article aims to review evidence of the potential role of TAAR1 in the pathophysiology and treatment of depression. Moreover, we briefly summarize the recent findings in the elucidation of behavioral and physiological properties of TAAR1 agonists both in clinical trials and preclinical animal studies. Collectively, these studies will provide a solid foundation for TAAR1 as a novel therapeutic target for depression.

## 1 INTRODUCTION

Depression is a serious illness, with more than 300 million cases worldwide, and is characterized by chronicity, recurrence, and a high suicide rate [1]. It is characterized by anhedonia, anxiety, pessimism, or even suicidal thoughts [2]. In addition to changes in mental state, cognitive dysfunction, and sleep disturbances are common symptoms of depressive disorders and may persist even after symptomatic remission [3, 4]. The disease imposes a considerable economic burden on patients and the social health care system, and some patients with major depressive disorder (MDD) even become incapacitated and need constant care [5]. In addition to a poor quality of life, numerous epidemiological studies have reported a close and bidirectional association between depression and cardiovascular disorders (CVDs) [6]. Research suggests that patients with depression are more likely to eventually develop CVD, indicating it is a robust risk factor for the development of CVDs in healthy subjects. Therefore, depression is a leading contributor to the global disease burden, making its effective treatment an urgent priority in modern psychiatry.

## 2 SEARCH STRATEGY AND SELECTION CRITERIA

We searched PubMed, Web of Science, Wolters Kluwer, Engineering Village 2, and search engines such as Google and Safari for publications in English from January 1, 2010, to July 31, 2024 with the keywords “depression”, “TAAR1”, “Ulotaront”, “neuropsychiatric disorders”, “antidepressant”, “signal pathway” and “mechanisms”. Furthermore, any publications that were not written in English and the impact factor of studies under 2.0 points were excluded from our review. Finally, some clinical cases and conference reports have been incorporated into the discussion sections that are pertinent to the theme of this review.

### 2.1 Pathogenesis of Depression

Depression is a complex disorder that spans cognitive, emotional, motivational, and physiological domains and has enormous effects on individual health. It is commonly suggested that early-life stress (ELS) is one of the etiological factors in the development of depression [7]. Epigenetic mechanisms have been proposed to mediate the lasting increase in the risk of depression following exposure to adverse life experiences [8]. Thus, this mental disorder results from a complex interaction between genetic and environmental factors. However, identifying the exact aetiology of depression is challenging, as patients with depression present with a constellation of symptoms that are unlikely to be explained by a single unifying mechanism.

A reduced hippocampal volume is the most common finding in neuroimaging studies of patients with depression. Several meta-analyses exploring the magnetic resonance imaging (MRI) volume of the hippocampus have revealed volume reductions of 4% to 10% in patients with depression [9, 10]. In addition, human postmortem studies have revealed a reduction in the hippocampal volume in depressed patients [11]. An increasing number of preclinical and clinical studies have highlighted that the pathophysiology of depression involves several mechanisms, including alterations in the serotonergic and glutamatergic systems, hypothalamic-pituitary-adrenal (HPA) axis abnormalities, increased levels of proinflammatory cytokines (neuroinflammation), decreased neurogenesis and neuroplasticity, and gut microbiota (GM) dysbiosis [12, 13]. Despite major advances in the field of neuroscience over the past few decades, the pathophysiology of depression has not been fully elucidated. Thus, a better understanding of the pathophysiological mechanisms underlying depression is particularly important for future therapeutic strategies.

### 2.2 First-line Treatments for Depression

The first-line treatments for depression are antidepressant medication (ADM), psychotherapy, and a combination of both. Psychological interventions are effective in the treatment of mild depressive episodes (mild-to-moderate depression) [14], have longer-lasting effects than antidepressant treatments do, and are preferred by most patients with postpartum depression. The psychological treatment with the greatest evidence base for depression is cognitive behavioural therapy (CBT) [15]. Intervention protocols vary and incorporate four core components: psychoeducation, self-monitoring, motivational interviewing, and problem-solving. In addition to psychotherapies, antidepressant medications, including selective serotonin reuptake inhibitors (SSRIs), serotonin-norepinephrine reuptake inhibitors (SNRIs), bupropion, and mirtazapine, constitute the mainstay of treatment for patients with moderate to severe depression [16]. Most currently available antidepressants target monoamine neurotransmitter function. Although great achievements have been achieved in the development of antidepressants, no significant advances in their antidepressant effectiveness or innovative mechanisms of action have been reported. For example, approximately one in three adults with MDD do not exhibit a clinical response to the current antidepressant medication [17], and even among respondents, a proper therapeutic effect may require weeks of treatment. In addition, severe side effects and adverse reactions limit the clinical use of these drugs. Thus, more effective, rapid-acting antidepressants with novel mechanisms of action are clearly needed.

## 3 TAAR1 AS A TARGET FOR THE DEVELOPMENT OF NOVEL ANTIDEPRESSANT DRUGS

### 3.1 Trace Amines and TAAR

**Fig. (1):**
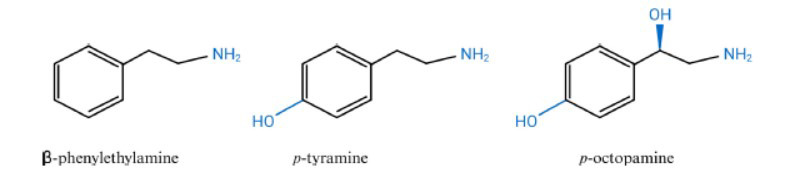
Structures of representative trace amines (TAs) [26]. The TAs PEA and TYP are synthesized from the decarboxylation of their precursor AAs by the AADC. OCT is synthesized from the hydroxylation of TYP by DA-β-decarboxylase. **Abbreviations**: TAs, trace amines; PEA, β-phenylethylamine; TYP, tyramine; AAs, amino acids; AADC, aromatic l-amino acid decarboxylase; OCT, octopamine; DA, dopamine.

Trace amines (TAs), such as β-phenethylamine (β-PEA), tyramine (TYR), tryptamine (TRP), and octopamine (OCT), as well as several other noncatechol amines, constitute a group of amines normally present in the mammalian brain at low nanomolar concentrations (Fig. **1**) that can modulate monoamine transmission [18, 19]. TAs are a family of endogenous compounds that are structurally similar to the biogenic amine neurotransmitters, such as serotonin (5-HT), norepinephrine (NE), and dopamine (DA). In addition to their structural similarity, TAs may have a role in the regulation of emotional behaviours, thoughts, mood, or perception along with these classical monoamine neurotransmitters [20, 21]. Alterations in functions of TAs are reported to involve in the aetiology of several psychiatric disorders, including MDD, schizophrenia, and anxiety states [22, 23]. A study by Davis *et al*. revealed that urinary excretion of TAs was significantly reduced in patients with depressive symptoms [23], whereas long-term administration of β-PEA, or l-phenylalanine (its precursor), significantly improved the affective state of patients with depression [24]. However, despite decades of research into their function, no specific receptors for trace amines (TAs) have been identified, which remains the greatest obstacle to advancing our understanding of these neurochemicals.

In 2001, Borowsky *et al*. reported that some members of a family of novel mammalian G protein-coupled receptors (GPCRs) presented high affinity for TAs [19]. This family of newly discovered receptors was subsequently renamed the trace amine-associated receptor (TAAR) family [25]. In vertebrates, 9 TAARs have been identified (TAAR1–9) [26]. They are predominantly expressed in the olfactory epithelium with the exception of TAAR1 [27]. There are 6 functional TAAR family members expressed in humans (TAAR1, 2, 5, 6, 8, and 9), with TAAR3, TAAR4, and TAAR7 subtypes being encoded by pseudogenes [28]. The discovery of TAARs has rekindled interest in investigating TAs as neurotransmitters or neuromodulators in the brain, although most of these receptors are still classified as orphan receptors.

### 3.2 TAAR1

**Fig. (2):**
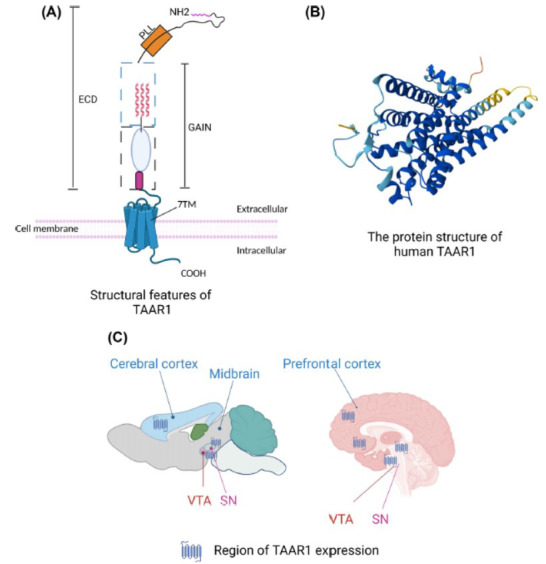
Structural features of TAAR1. (**A**) A schematic depicts the general structural organization of TAAR1. TAAR1 is a GPCR that consists of a large N-terminal ECD followed by 7TMs. The ECD contains multiple cell adhesion protein domains followed by a GAIN domain. This figure was adapted from our previously published article [95]. (**B**) The protein structure of human TAAR1 was created with the AlphaFold Monomer v2.0 pipeline. (**C**) A schematic drawing of the mouse (left panel) and human (right panel) brain shows the primary regions in which TAAR1 is expressed. TAAR1 is highly expressed in the VTA and DRN in rodents [29, 30]. In addition, TAAR1 expression has also been found in pyramidal neurons of layer 5 of PFC [32]. This figure was adapted from an article previously published by Halff *et al*. [49]. The distribution of TAAR1 in the human brain is inferred from its expression in rodents, but more research is needed for verification. The figure was generated using BioRender (Agreement number: HF27UVYNU5). Abbreviations: VTA, ventral tegmental area; SN, substantia nigra; ECD, extracellular domain; TMs, seven transmembrane domains. GAIN, GPCR autoproteolysis-inducing; TAAR1, trace amine-associated receptor 1.

TAAR1 is a GPCR and one of the best-studied receptors of TAs (Fig. **2**). It is expressed in the mammalian brain at a low level and is broadly expressed in monoaminergic systems (Fig. **2**), including the ventral tegmental area (VTA), substantial nigra (SN), and dorsal raphe nucleus (DRN) [29, 30]. In addition to being expressed in subcortical areas, it is also observed in cortical areas, especially in layer V pyramidal neurons of the prefrontal cortex (PFC) [31, 32]. Although the distribution of TAAR1 in the brain is well-explored, its cell type-specific and regional expression is an ongoing topic of debate due to the expression at a relatively low level in the mammalian brain and the lack of suitable antibodies [33], and additional studies are needed to elucidate its expression in both cell systems and *in vivo*. To date, TAAR1 has been demonstrated to negatively modulate monoamine transmission and be involved in many mental diseases, including depression, anxiety, schizophrenia, and sleep disorders [34, 35]. In recent years, a growing number of studies have demonstrated that TAAR1 plays an important role in the treatment of depression, demonstrating antidepressant properties [36]. Interestingly, TAAR1 agonists also show valid properties for the treatment of depression [37, 38]. In this review, we focus on the role of TAAR1 in regulating depression-like behaviours and the related underlying neural mechanisms.

### 3.3 Downstream Targets of TAAR1

Although the downstream targets of TAAR1 have not been fully understood, some studies have revealed important findings about TAAR1-mediated signalling. Due to its localization in different cellular compartments, TAAR1 may activate distinct signalling pathways [39]. For example, Zheng *et al*. reported that oral administration of natto for 12 weeks ameliorated cognitive decline by activating the TAAR1-mediated calcium/calmodulin-dependent protein kinase II (CaMKII)/cAMP response element-binding protein (CREB)/ brain-derived neurotrophic factor (BDNF) signalling pathway in the hippocampus of senescence-accelerated mouse prone 8 (SAMP8) mice [40]. TAAR1 can bind to Gα_s_ proteins and, when stimulated, triggers the accumulation of intracellular cyclic adenosine monophosphate (cAMP) *via* the activation of adenylyl cyclase (AC), followed by protein kinase A (PKA) and protein kinase C (PKC) activation, as well as their downstream signalling pathways [26, 28, 29]. Endogenous intracellular TAAR1 also binds to a G-protein α subunit, G_13_, which is commonly associated with ras homology family member A (RhoA) guanosine triphosphatase (GTPase) activation [39]. In addition, increased TAAR1 expression on activated lymphocytes is capable of mediating the activation of the CREB and nuclear factor of activated T cells (NFAT) signalling pathways along with concurrent changes in phosphorylated PKA (pPKA) and PKC (pPKC) [41]. Furthermore, TAAR1 stimulates inwardly rectifying K(+) channels, leading to an outwards K(+) current that reduces the excitability of DRN and VTA neurons [42, 43]. Moreover, it signals *via* a G protein-independent, β-arrestin2 (βArr2)-dependent pathway involving the protein kinase B (AKT)/glycogen synthase kinase 3 (GSK-3) β signalling cascade, an important pathway in many dopamine (DA)-mediated actions [31].

**Fig. (3):**
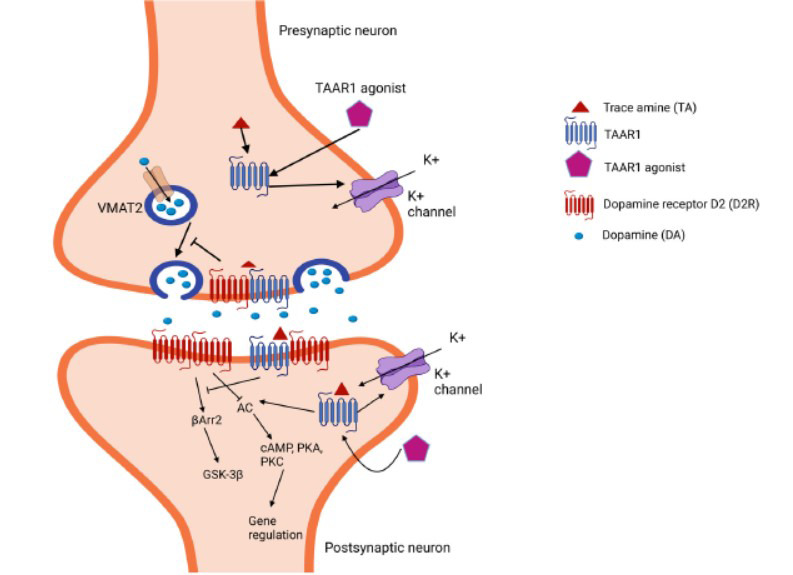
The molecular pathways of the activation of TAAR1 at a DA synapse. Endogenous TAs activate TAAR1, resulting in AC activation and downstream stimulation of PKA/PKC. In the postsynaptic domain, TAAR1 counteracts the normal effects of D2R activation. TAAR1 signals *via* a G protein-independent, βArr2 pathway involving the protein kinase B (AKT)/ GSK-3β signalling cascade. Heterodimerization of TAAR1 prevents βArr2 recruitment and blocks GSK3β activity [52, 53]. Furthermore, TAAR1 activates G protein-coupled inwardly rectifying potassium channels, leading to an outwards K current [29, 44]. The figure was adapted from Halff *et al*. [49] and generated using BioRender (Agreement number: UF27UVYSDL). AC, adenylyl cyclase; TAAR1, trace amine-associated receptor 1; TAs, trace amines; PKA, protein kinase A; PKC, protein kinase C; βArr2, β-arrestin2-dependent; GSK-3, glycogen synthase kinase 3; VMAT2, vesicular monoamine transporter 2.

Similarly, other studies have shown that TAAR1 is activated by TAs, such as TYR and β-PEA, which are normally expressed in the mammalian brain at low concentrations [26, 44]. Moreover, a wide range of endogenous and exogenous molecules activate TAAR1, including DA, catecholamine metabolites, amphetamine analogues [45], methamphetamine (MA), norepinephrine, 5-HT, and some of their metabolites [46-48]. The interaction of TAAR1 with dopamine receptor D2 (D2R) has been shown to exert functional effects on both presynaptic and postsynaptic modes of action [49]. When coexpressed with D2R, TAAR1 heterodimerizes with D2R to regulate D2R function at the plasma membrane [50]. Activated TAAR1 potentiates its inhibitory role through heterodimerization with presynaptic dopamine D2 autoreceptors (D2ARs) [51]. Specifically, TAAR1 improves the ability of D2ARs to reduce tyrosine hydroxylase (TH) activities by blocking its phosphorylation, which reduces L-dihydroxy-phenylalanine (L-DOPA) synthesis and increases the ability of D2ARs to reduce DA release [51]. In the postsynaptic domain, TAAR1 counteracts the normal effects of D2R activation (Fig. **3**). Heterodimerization with TAAR1 prevents βArr2 recruitment and leads to GSK3β inactivation [52, 53]. Notably, many studies have shown that aberrant activities of GSK3β have multiple negative effects and are related to the severity of depressive symptoms, including impaired neurogenesis [54]. Therefore, these findings further suggest the potential for TAAR1 as a powerful tool to ameliorate depression.

### 3.4 Synthetic TAAR1 Agonists

Activated TAAR1 may function as an antidepressant drug by improving NE and 5-HT levels in the synaptic cleft, which makes TAAR1 an attractive target for the treatment of reduced levels of neurotransmitters in the brain, as observed in patients with depression. Over the past decade, studies of selective TAAR1 agonists have made statistically significant progress in understanding TAAR1 function at the behavioural level. What's more, several TAAR1 agonists have been demonstrated as novel pharmaceuticals for depression treatments (Table **1**). These agonists have been tested in preclinical animal experiments that represent reliable models for modelling the pathophysiology of depression. Strikingly, several of these compounds (TAAR1 agonists) are now entering human clinical trials [38]. Therefore, using synthetic TAAR1 agonists in depression has further deepened our understanding of the functional mechanism of TAAR1.

## 4 THE SUPPRESSING EFFECTS OF TAAR1 AGONISTS ON CHRONIC STRESS-INDUCED BEHAVIOURS, SUCH AS DEPRESSION

### 4.1 Potential Antidepressant Action of SEP-363856 (a Dual TAAR1 and 5-HT1A Receptor Agonist) in Humans and Rodents

In consideration of the enriched expression of TAAR1 in the amygdala [29], an evolutionarily conserved core structure involved in emotion processing and one of the key regions of interest in affective neuroscience [55], it might play a crucial role in emotional regulation. The relevancy between TA deficiency and depression [23, 56] indicates that the activation of TAAR1 might function to stabilize emotional fluctuations and maladaptive mood and contribute to the antidepressant-like effects. In support of this hypothesis, current research suggests that several TAAR1 agonists have useful properties for antidepressant therapy [57].

**Fig. (4):**
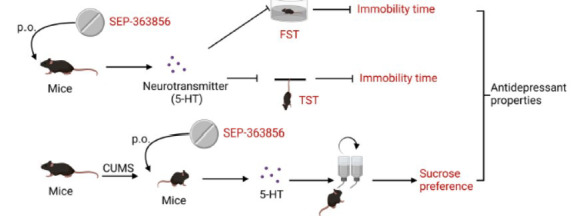
A TAAR1 agonist (SEP-363856) has a significant antidepressant-like effect on mice [37]. Compared to vehicle, SEP-363856 (a single oral administration) at 0.3, 1, or 10 mg/kg significantly reduced the immobility time of mice in the FST, suggesting obvious antidepressant effects of SEP-363856 [37]. In the TST, compared with the vehicle (Veh), only 0.3 mg/kg SEP-363856 significantly decreased the immobility time [37]. Moreover, SEP-363856 (15 mg/kg) reversed the CUMS-induced significant decrease in SPT when compared with the CUMS-Veh group, indicating antidepressant-like efficacy [37]. These results indicate that SEP-363856 could exhibit an important role in depression. The figure was generated using BioRender (Agreement number: TS27UVYUI7). SPT, sucrose preference test.

Ren *et al*. reported that a TAAR1 agonist (SEP-363856) had a significant antidepressant-like effect on mice (Fig. **4**) [37]. Interestingly, compared to vehicle, a single oral administration of SEP-363856 at 0.3, 1, or 10 mg/kg significantly reduced the immobility time of mice in the FST, suggesting obvious antidepressant effects of SEP-363856 [37]. The FST is a behavioural assay for the study of depressive-like behavior in rodents and is sensitive to all major classes of marketed antidepressant drugs [58]. In the TST, compared with the vehicle (Veh), only 0.3 mg/kg SEP-363856 significantly decreased the immobility time [37]. Moreover, SEP-363856 (15 mg/kg) and duloxetine independently and synergistically reversed CUMS-induced significant declines in the sucrose preference test (SPT) compared with the CUMS-Veh group, also indicating antidepressant-like efficacy [37]. These data suggest that SEP-363856 could play a certain role in depression, and its antidepressant properties were shown for the first time in the TST and SPT [37]. Notably, SEP-363856 is a dual 5-HT1A partial agonist and TAAR1 agonist that induces a significant inhibitory effect *via* the 5-HT1A receptor in serotonergic neurons in the DRN both *in vivo* and *in vitro* [59, 60]. Therefore, we believe that the antidepressant properties of SEP-363856 may occur *via* the increased potency of 5-HT1A and the reduced desensitization rate of 5-HT1A in the DRN [61]. Additional larger-scale clinical studies are needed to investigate the antidepressant potential of SEP-363856 as a new generation of antidepressant compared to traditional antidepressant therapy.

Consistent with these findings, Dedic and colleagues reported that, compared to vehicle, SEP-363856 (a single oral administration) at 1, 3, and 10 mg/kg significantly reduced the immobility time of mice in the FST [62], indicating it might also have antidepressant-like activities without a clear dose-dependent effect. Moreover, SEP-363856 also decreased rapid eye movement (REM) sleep (3 and 10 mg/kg), increased the latency to REM sleep (10 mg/kg), and increased the cumulative wake (W) time (10 mg/kg) in rats in a dose-dependent manner [62]. Depression is characterized by an increase in REM density and a decrease in REM sleep latency [63]. Collectively, these results suggest that SEP-363856 exerts its antidepressant effect through the direct modulation of the 5-HT1A receptor and TAAR1, indicating that SEP-363856 might represent a promising candidate for the treatment of depression and has the potential to treat other psychiatric disorders.

Although SEP-363856 has been shown to suppress REM sleep in rodents [62], whether SEP-363856 affects REM sleep in humans is unclear. Recently, in a randomized, double-blind, placebo-controlled, 2-way crossover study of the effects of administration of SEP-363856 (single oral) at 10 and 50 mg doses on REM sleep in healthy male subjects [60], Hopkins *et al*. found that SEP-363856 (50mg) significantly reduced the REM duration and increased the latency to REM in healthy subjects compared with the placebo, whereas the single dose of SEP-363856 (10mg) had a lower REM-suppressive effect (it increased the REM latency but did not change the REM duration as compared with the placebo) [60]. Moreover, at dosages of 10mg and 50mg, it both showed favourable clinical tolerability and safety in patients [60]. Therefore, we believe that the REM-suppressing effects of SEP-363856 are likely driven by the activation of 5-HT1A and/or TAAR1 receptors because some studies have shown that it does not increase monoamine release in mice [62]. Notably, SEP-363856 increased the cumulative wake (W) time in rats without altering cumulative nonrapid eye movement (NREM) sleep [62]. In comparison, Hopkins *et al*. did not observe apparent differences in total sleep time during SEP-363856 treatment, and a small increase in the amount of time spent in NREM stage 2 and stage 3 (slow wave) sleep was observed in subjects treated with SEP-363856 (50mg) compared with those treated with the placebo [60]. These contradictory results are attributed to the differences in certain measures of sleep electroencephalography (EEG) across species, and clinical trials are needed in the future to determine the utility of SEP-363856 for REM sleep.

However, another post hoc exploratory analysis designed by Feemster *et al*. was reported to evaluate the effect of SEP-363856 on quantitative REM sleep without atonia (RSWA) [64], which is thought to be a prodromal or subclinical state of the disease and is a main polysomnographic (PSG) feature of REM sleep behaviour disorder (RBD) [65]. Research has indicated that RSWA is positively associated with depressive symptoms in patients with narcolepsy type 1-REM sleep behaviour disorder (NT1-RBD) [66]. Feemster *et al*. reported that a single dose of SEP-363856 (50 mg, but not 10 mg) had significant REM-suppressing effects on young, healthy adult men (aged 19-35) [64]. Furthermore, at a dosage of 50 mg, SEP-363856 significantly reduced RSWA levels in healthy adult men, especially in subjects with higher baseline RSWA levels as compared with those treated with the placebo, whereas a 10 mg dose of SEP-363856 had no clear effect on RSWA levels [64]. These differences may be caused mostly by small sample sizes, and thus, further studies are needed to investigate the potential efficacy of SEP-363856 for RBD.

### 4.2 RO5263397, a Selective TAAR1 Partial Agonist

Currently, TAAR1 agonists have been comprehensively studied and are highly important compounds for the treatment of mental disorders. In addition to SEP-363856, RO5263397, a partial agonist of TAAR1, has shown potential antidepressant activity in rodents.

**Fig. (5):**
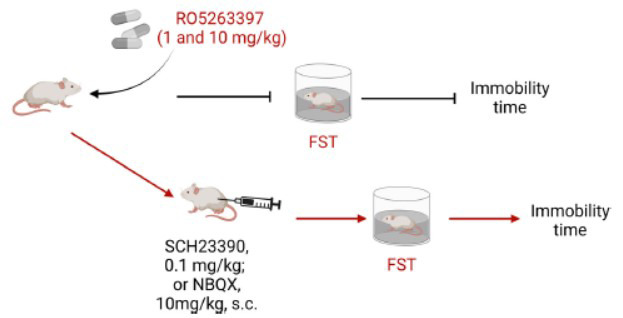
RO5263397, a partial agonist of TAAR1, has shown potential antidepressant activity in rodents [67]. Compared with the vehicle, RO5263397 at 1 and 10 mg/kg (p.o.) significantly reduced immobility in rats [67]. Moreover, the reduced immobility in rats treated with RO5263397 (1 mg/kg) was blocked by both the D1R antagonist SCH23390 (0.1 mg/kg, subcutaneous injection, s.c.) and the alpha-amino-3-hydroxy-5-methyl-4-isoxazolepropionic acid (AMPA) antagonist NBQX (10 mg/kg, s.c.) [67], indicating that the antidepressant properties of RO5263397 in the FST in rats involve the AMPA glutamate receptor and the D1R. The figure was generated using BioRender (Agreement number: VB27UVYWB7).

An early study by Espinoza *et al*. indicated that 1 and 10 mg/kg (p.o.) RO5263397 significantly reduced immobility in rats compared with the vehicle, and the effect was similar in magnitude to that of fluoxetine (10 mg/kg, p.o.) [67]. In the same study, the authors also showed that the reduced immobility in rats treated with RO5263397 (1 mg/kg) was blocked by both the D1R antagonist SCH23390 (0.1 mg/kg, subcutaneous injection, s.c.) and the alpha-amino-3-hydroxy-5-methyl-4-isoxazolepropionic acid (AMPA) antagonist NBQX (10 mg/kg, s.c.) [67], indicating that the antidepressant properties of RO5263397 in rats performing the FST involve the AMPA glutamate receptor and the D1R (Fig. **5**). However, in another study, the same authors reported that TAAR1 directly interacted with D2Rs but not D1Rs [50]. Moreover, the interaction of TAAR1 with D2Rs influences the TAAR1-mediated effect on the dopamine system, especially the pronounced supersensitivity of postsynaptic D2Rs in the striatum and increased D2-dependent locomotor activation [31]. Therefore, the antidepressant activity of RO5263397 might not be mediated by a direct interaction of TAAR1/D1Rs but is likely regulated by a functional effect on D1Rs located in cortical areas that have been linked to the antidepressant action. Another preclinical study by Sun *et al*. revealed the possible molecular mechanism by which the D1R in the medial prefrontal cortex (mPFC) of mice mediates neuroprotective effects of RO5263397 on chronic social defeat stress (CSDS)-induced social and cognitive deficits [68]. Notably, a D1R antagonist (SCH23390) blocked anti-depressant effects of RO5263397 on chronic stress-induced social and cognitive dysfunction [68], suggesting a functional interaction between TAAR1 and D1R. These findings are inconsistent with those of previous studies [50], and this discrepancy might be due to the different stress paradigms used and the use of different animal models.

Similarly, Zhang *et al*. reported that RO5263397 (1.5mg/kg, i.p.) was uniquely useful for treating MDD-related cognitive dysfunction [69], part of the symptomatology of depressive disorders [70]. In their study, the TAAR1 mRNA level was significantly decreased in the mPFC of CSDS-exposed mice, whereas RO5263397 treatment reversed CSDS-induced social avoidance and cognitive deficits by improving the length and density of dendrites of pyramidal neurons in the mPFC of mice [69]. Furthermore, RO5263397 reversed the imbalance in the excitatory-inhibitory (E/I) ratio in the prelimbic mPFC (PrL) of CSDS-exposed mice [69], which is an important molecular pathological feature of MDD. For this reason, we believe that RO5263397 treatment ameliorated impaired cognitive function in CSDS-exposed mice, which might be related to the regulation of the unbalanced neural E/I ratio in the PrL. Similarly, in another study, the same authors reported that RO5263397 treatment (1.5mg/kg, i.p.) mitigated the detrimental effects of CSDS on hippocampal plasticity and cognitive function [36], indicating that RO5263397 plays an essential role in reducing detrimental effects of chronic stress on impairing neurogenesis and cognition. In summary, the development of partial TAAR1 agonist-based drugs has provided a new therapy for depression, especially for improving chronic stress-induced cognitive deficits. However, a recent study showed that acute treatment with RO5263397 did not alter the excitability of 5-HT neurons in the DRN or the excitability of DA neurons in the VTA *in vivo* when evaluated *via* an electrophysiological method [71]. This finding was not consistent with those of previous studies, which reported that RO5263397 increased the firing frequency of VTA DA and DRN 5-HT neurons in slices from wild-type (WT) mice [72]. A possible explanation is that RO5263397-mediated blockade of TAAR1 receptors in other brain regions, such as the mPFC, putatively neutralizes the direct effect of 5-HT neurons on the DRN *in vivo*, whereas the blockade of TAAR1 receptors expressed in the DRN directly stimulates the excitability of 5-HT neurons *in vitro*. However, this mechanism remains largely understudied and deserves further exploration and investigation.

Recently, several animal studies have been conducted to evaluate the wake-promoting effects of RO5263397 and its potential to treat sleep disorders [73]. RO5263397 (1 and 3 mg/kg, p.o.) dose-dependently increased the cumulative wake time and decreased the cumulative times in NREM and REM sleep, although it did not alter the latencies to REM and NREM sleep [72]. Another study also showed that RO5263397 (0.3 and 1 mg/kg, p.o.) promoted wakefulness and suppressed REM and NREM sleep in WT mice, whereas this effect was absent in TAAR1 knockout (KO) mice [74]. Furthermore, RO5263397 produced wake-promoting, and NREM and REM sleep-inhibiting effects on WT mice, and these effects were potentiated in overexpressing (OE) mice but attenuated in KO mice [73].

In conclusion, these findings suggest that the selective partial agonist of TAAR1, RO5263397, might represent a new drug to address cognitive deficits and sleep disorders in patients with depression. However, further clinical studies are needed to explore the possible mechanisms underlying the antidepressant effects of TAAR1 activation.

### 4.3 The Effect of the Selective TAAR1 Partial Agonist RO5203648 on Depression

RO5203648, the first selective TAAR1 partial agonist to be identified, has shown high affinities and potencies for primate and rodent TAAR1 expressed *in vitro* [75]. The first evidence that RO5203648 possesses favourable pharmacokinetic properties was obtained from a previous report by Revel and colleagues in which they indicated clear antidepressant-like activities of RO5203648 in rodents and monkeys [75]. Importantly, RO5203648 increases the firing frequency of 5-HT and DA neurons in mouse brain sections [75], similar to a TAAR1 antagonist EPPTB [42]. In addition, compared with the vehicle, RO5203648 dose-dependently (10 and 30 mg/kg, p.o.) reduced the immobility time of rats, suggesting that RO5203648 has antidepressant-like properties [42]. In particular, RO5203648 (10 mg/kg, p.o.) significantly promoted wakefulness and increased the latency to NREM and REM sleep in rats, whereas it reduced the cumulative time in REM and NREM sleep [42]. These data suggest that RO5203648 displayed clear beneficial activity in the FST paradigm, fully supporting its therapeutic potential in depression.

Methamphetamine (METH) is a highly addictive psychotropic compound in the world that is highly toxic to the central nervous system (CNS), and chronic use and/or high-dose administration of METH frequently leads to psychiatric disorders, including depression, anxiety, and schizophrenia, of which depression has a relatively high incidence [76]. Cotter *et al*. measured the DA outflow from the nucleus accumbens (NAc) using microdialysis *in vivo* and found that RO5203648 (5 mg/kg, i.p.) reversed METH-induced a significant increase in DA levels in the first 20 minutes after the administration of METH in rats, whereas it did not alter METH-mediated DA efflux and uptake inhibition in striatal synaptosomes *in vitro* [77], indicating that the regulatory effect of RO5203648 on the behavioural effects of METH is unlikely to depend on direct, local actions at the dopamine transporter (DAT). Therefore, these findings indicate that RO5203648 can regulate DA activities and has the potential to act as an important compound to modulate METH-induced dysregulation of DA in patients with depression.

### 4.4 RO5166017, a TAAR1 Full Agonist on Depression

In 2011, Revel and coworkers engineered a selective TAAR1 agonist, RO5166017, which exhibited high potencies and efficacies for human, cynomolgus monkey, mouse, and rat TAAR1 stably expressed in HEK293 cells [61]. *In vitro* (in mouse brain slices), it inhibited the firing rate of DA neurons in the 5-HT and VTA neurons in the DRN [61]. Notably, RO5166017 had no effect on either electrophysiology or behaviour in *Taar1^−/−^* mice [61], indicating that functional TAAR1 is required to mediate the effects of RO5166017. In addition, some studies demonstrated that RO5166017 could effectively block cocaine relapse in the extinction/reinstatement paradigm [78]. Depression, a key symptom of cocaine withdrawal syndrome in addicted individuals, is considered the main factor that precipitates relapse in individuals with chronic cocaine addiction [79]. RO5166017 decreases yohimbine‐induced reinstatement of cocaine‐seeking behaviour [78], suggesting that a TAAR1 agonist (RO5166017) is an effective compound that can attenuate cocaine addiction and relapse. Yohimbine is an alpha-2 receptor antagonist and is frequently used as a chemical stressor in animal studies of stress-induced relapse because of its robust and reliable nature [80, 81]. A reasonable explanation is that RO5166017 might counteract the yohimbine‐induced stress‐like state, as a previous study by Revel *et al*. revealed that, compared with WT/Veh, RO5166017 at a higher dose (1 mg/kg) reduced the basal body temperature (*T*_b_) [61]. Due to the antistress properties of RO5166017, we believe that RO5166017 inhibited but did not potentiate the effect of yohimbine.

Another preclinical study showed that a microinjection of RO5166017 (5.0 μg/0.5 μl/side) into the PrL and VTA of rats reduced both cue- and drug-priming-induced cocaine-seeking behaviour [82], which kept in line with previous studies suggesting that the use of RO5166017 reduced the firing frequency of DA neurons in the VTA [61]. Moreover, microinjection with RO5166017 into the NAc core (NACc) and shell (NACs) inhibited cue- and drug-induced cocaine-seeking behaviours, respectively [82]. The NAc has two major components, the NACc, and NACs, which have different inputs and outputs, indicating that they contribute differently to goal-directed behaviours [83]. Previous results indicated that intra-NACc administration of muscimol+baclofen (Mus+Bac) weakened conditioned cue-induced reinstatement of cocaine seeking, while (Mus+Bac) administration (intra-NAC) did not alter the same behaviour compared to the vehicle pretreatment [84], indicating that the core and shell regions of the NAc might play different roles in stimulus-induced motivated behaviour. However, no significant changes in cocaine-seeking behaviours were observed when RO5166017 was microinjected into the substantia nigra (SN), basolateral amygdala (BLA), infralimbic cortex (IL), or central amygdala (CeA) [82]. Notably, RO5166017 injection in the amygdala is not involved in the reinstatement of cocaine-seeking behaviour; a reasonable explanation is that the TAAR1 mRNA is expressed at lower levels in the amygdala than in the NAc [82].

In general, these findings elucidate a novel mechanism by which RO5166017 regulates drug-induced reinstatement of cocaine seeking and further suggest that RO5166017 is a promising TAAR1 agonist for the prevention of cocaine relapse.

### 4.5 The Effect of RO5256390 (a TAAR1 Full Agonist) on Depression

Like RO5166017, RO5256390 is also a selective and highly potent TAAR1 agonist that is well-suited for *in vivo* investigations in primates and rodents [72]. RO5256390 stimulation leads to an increase in cyclic adenosine monophosphate (cAMP) levels of 79-107%, which is comparable to that achieved by PEA (set at 100%) [72]. Moreover, it decreases the firing frequency of DRN 5-HT and VTA DA neurons, whereas this effect is not detected in slices from *Taar1^−/−^* mice [72]. Notably, compared with the vehicle, none of the investigated doses of RO5256390 significantly affected the immobility time of rats in the FST [72], and RO5256390 did not affect sleep-wake activities in rats [72], indicating its potential antidepressant-like properties with lower intrinsic efficacy [72].

Grinchii *et al*. evaluated the effects of chronic and acute administration of RO5256390 on the *in vivo* excitability of central monoamine-secreting neurons in rats [71]. They reported that RO5256390, with acute administration, inhibited the firing activity of DA neurons in the VTA and 5-HT neurons in the DRN in a dose-dependent manner [71]. Moreover, RO5256390 (chronic administration) elevated the firing rate of DA neurons but did not alter the firing rate of 5-HT neurons, whereas it stimulated the burst-mode firing of 5-HT and DA neurons [71]. We speculate that chronic RO5256390 treatment might result in desensitization of 5-HT1A receptors, and further investigations are needed to support this hypothesis. These findings suggest that the antidepressant effects of TAAR1 ligands might be mediated by the modulation of the excitability of 5-HT and DA neurons. More importantly, acute and chronic administration of RO5256390 might result in distinct physiological changes, which ultimately influence the pharmacological properties of this compound at the behavioural level.

RO5256390 has also been reported to exhibit an important role in treating narcolepsy. RO5256390 profoundly reduced REM sleep but not the amount of wakefulness or NREM sleep in WT mice, whereas this effect (inhibition of REM sleep) was eliminated in Taar1 KO mice [74], indicating it may be a promising TAAR1 agonist for the treatment of sleep-related disorders.

In summary, the present findings showed that increasing 5-HT and DA neurotransmission *via* the modulation of 5-HT neurons in the DRN and DA neurons in the VTA might be involved in the antidepressant effects of a TAAR1 agonist (RO5256390). Notably, investigations into the effects of RO5256390 on the mechanisms of depression-like behaviours are still in their infancy, with most of the available evidence obtained from a few studies. This hypothesis should be tested in a large number of studies, including the assessment of clinical effects in the heterogeneity among human populations.

### 4.6 The Effect of a TAAR1 Agonist (o-PIT) on Depression

The thyroxine derivative o-phenyl-3-iodotyramine (o-PIT) behaves as an agonist for the activation of TAAR1 and has powerful efficacy and potency for hypothermia induction [85]. A recent study revealed the molecular mechanisms of the potential antidepressant activity of o-PIT [86]. O-PIT dose-dependently (20 and 40 mg/kg, i.p.) decreased the core temperature of WT and TAAR1-KO mice (the effect was less pronounced on TAAR1-KO mice), whereas this effect was blocked by the α2-adrenoceptor antagonist RX821002 [86], supporting a role for TAAR1 in the mediation of hypothermia elicited by o-PIT. In addition, a microdialysis probe revealed that the administration of o-PIT at a dosage of 10 mg/kg produced a sustained increase in DA levels in the mPFC of WT mice but not TAAR1-KO mice [86], whereas it did not alter the levels of 5-HT or noradrenaline (NA) [86], suggesting that TAAR1 is involved in DA release in the mPFC stimulated by o-PIT. Interestingly, classical antidepressants such as venlafaxine, citalopram, and reboxetine increase 5-HT levels in both WT and TAAR1-KO mice [86], indicating that their antidepressant effects do not involve TAAR1. Furthermore, the duration of immobility was dose-dependently reduced by o-PIT (20 and 40 mg/kg) in WT mice but was not affected in TAAR1-KO mice, suggesting that the antidepressant action of o-PIT was mediated by TAAR1 [86]. In contrast, no significant difference in immobility time was observed between WT and TAAR1-KO mice after the administration of several commonly used antidepressants [86], further supporting the hypothesis that the neurochemical and behavioural actions of the antidepressants used in clinical treatment do not involve TAAR1. These results indicate that o-PIT may be a useful tool for investigating TAAR1-mediated antidepressant actions, particularly those related to the DA system.

## 5 THE ROLE OF EPPTB IN NEURONAL EXCITABILITY

To date, the selective and highly potent mouse TAAR1 (mTAAR1) antagonist EPPTB (N-(3-ethoxy-phenyl)-4-pyrrolidin-1-yl-3-trifluoromethyl-benzamide) has been the only one identified [42]. EPPTB blocked the p-tyramine (p-tyr, a nonselective TAAR1 agonist)-mediated reduction in the firing frequency of DA neurons in the VTA in WT mice but not in *Taar1*^−/−^ mice [42], indicating high selectivity. A recent study revealed that EPPTB suppressed the hyperexcitability of pyramidal neurons in hippocampal slices from kainic acid (KA)-induced mice [87]. This type of neuronal damage induced by overexcitation has been proposed to be involved in some psychiatric conditions, including depression [88]. Based on the evidence mentioned above, we believe that TAAR1 might be a potential drug target for individuals with depression. Indeed, due to the poor *in vivo* pharmacokinetic properties of EPPTB and its inability to cross the BBB [89], many more clinical trials with larger sample sizes are needed to confirm its efficacy in the future.

In contrast, another study demonstrated that EPPTB prevented 3-iodothyronamine (T1AM)-induced neuroprotection and the activation of protein kinase A (PKA) and protein kinase B (AKT) against KA toxicity [90]. Interest in T1AM, an active thyroid hormone metabolite that affords protection from excitotoxic damage, is growing [91]. Notably, in the presence of EPPTB, the neuroprotection offered by T1AM was reduced, indicating that TAAR1 inhibition by EPPTB impaired the mechanism of T1AM-mediated neuroprotection.

## CONCLUSION

Depression is a common psychiatric disorder associated with major public health implications and is ranked second in terms of the global burden of disease [92]. Although studies of its pathophysiology have focused mainly on 5-HT and serotonergic neurotransmission for over sixty years, the exact role of serotonin in depression pathophysiology is still unclear.

As the most well-characterized receptor in the TAAR family, TAAR1 has been shown to be involved in dopaminergic, serotonergic, and glutamatergic transmission and exhibits an important role in several psychiatric disorders [93]. Accumulating evidence indicates that TAAR1 is uniquely positioned to directly regulate the firing and release of DA and 5-HT neurons [71], which has profound implications for elucidating the pathophysiology of depression and for developing more effective treatment strategies for this condition that are strongly linked to the dysfunction of monoamine transmission. To date, the majority of published studies on the function of TAAR1 in depression have analysed the roles of TAAR1 agonists, which are the most frequently studied drugs targeting this receptor and are already in clinical trials as antidepressants [94]. The antidepressant effects of TAAR1 agonists are widely involved in the modulation of serotonergic and dopaminergic neuron firing through distinct mechanisms [59]. In addition, TAAR1 agonists have wake-promoting and cognitive-enhancing properties, but these effects are eliminated in Taar1 KO mice [69, 74]. Currently, clinical studies of the use of TAAR1 agonists for the treatment of depression are scarce but warranted. Based on these findings, changes in the neural activity of the brain during the blockade of TAAR1 seem to be extremely intriguing and promising [90].

In conclusion, the literature reviewed here strongly indicates an important role for TAAR1 in the pathophysiology of depression, and its agonists might be promising novel pharmacological treatments for depression. However, a comprehensive understanding of the underlying mechanisms by which TAAR1 exerts its potential antidepressant effects is lacking. A large number of studies are needed to fully elucidate downstream targets that are recruited by TAAR1 under physiological or nonphysiological conditions, as well as their pharmacokinetic/dynamic profiles.

## AUTHORS' CONTRIBUTIONS

The author confirms sole responsibility for the following: study conception and design, data collection, analysis and interpretation of results, and manuscript preparation.

## Figures and Tables

**Table 1:** Summary of preclinical findings on the effects of TAAR1 agonists on depression.

**TAAR1 Agonists**	**Dosage**	**Mechanism**	**References**
SEP-363856	A single oral administration of SEP-363856 at 0.3 mg/kg in the FST and TST or 15 mg/kg in the SPT	SEP-363856 showed antidepressant-like effects in depressive-like behavior in mice	[37]
RO5263397	Administered intraperitoneally (i.p). at a dosage of 1.5 mg/kg	The administration of RO5263397 reinstated the behavioral alterations and changes of structural plasticity in DG granule cells in CSDS-induced mice	[36]
RO5263397	0.3 and 1.0mg/kg, p.o.	Improvement in the sleep-wake cycle	[73]
TAAR1 full agonist RO5256390 and partial agonist RO5263397	10 mL/kg, p.o.	RO5256390 reduced REM sleep in WT mice, and RO5263397 also promoted wakefulness and suppressed NREM sleep	[74]
RO5263397	5.6 mg/kg, i.p.	Reduced the increased impulsivity induced by acute discontinuation from prolonged methamphetamine treatment	[96]
RO5256390	10mg/kg, i.p.	Has no effects on depressive-like behavior (FST) in rats	[97]
RO5263397	10 or 30mg/kg, p.o.	Has antidepressant-like effect in FST (rat)	[72]
Ulotaront (SEP-363856)	1, 3, and 10 mg/kg, i.p.	Has modest antidepressant effects in the mouse FST	[62]
Ulotaront (SEP-363856)	p.o.	Test its safety and efficacy in the treatment of major depressive disorder	[38]
o-PIT	20, 40 mg/kg, i.p.	Reduced the time of immobility in WT mice	[86]

**Abbreviations**: FST: forced swim test; TST: tail suspension test; SPT: sucrose preference test; CSDS: chronic social defeat stress; REM: rapid eye movement; WT: wildtype; NREM: non-rapid eye movement; o-PIT: o-phenyl-iodotyramine.

## LIST OF ABBREVIATIONS

AC: Adenylyl Cyclase
ADM: Antidepressant Medication
BDNF: Brain-derived Neurotrophic Factor
β-PEA: β-phenethylamine
CVDs: Cardiovascular Disorders
CBT: Cognitive Behavioural Therapy
cAMP: Cyclic Adenosine Monophosphate
DA: Dopamine
DAT: Dopamine Transporter
DRN: Dorsal Raphe Nucleus
EEG: Electroencephalography
GM: Gut Microbiota
HPA: Hypothalamic-pituitary-adrenal
IL: Infralimbic Cortex
MRI: Magnetic Resonance Imaging
MDD: Major Depressive Disorder
NE: Norepinephrine
OCT: Octopamine
PSG: Polysomnographic
PFC: Prefrontal Cortex
PKA: Protein Kinase A
SSRIs: Selective Serotonin Reuptake Inhibitors
SNRIs: Serotonin-norepinephrine Reuptake Inhibitors
SN: Substantia Nigra
TAs: Trace Amines
TRP: Tryptamine
TYR: Tyramine
TH: Tyrosine Hydroxylase
VTA: Ventral Tegmental Area
